# Chalcone Scaffold in Anticancer Armamentarium: A Molecular Insight

**DOI:** 10.1155/2016/7651047

**Published:** 2016-01-03

**Authors:** Manik Das, Kuntal Manna

**Affiliations:** Department of Pharmacy, Tripura University (A Central University), Suryamaninagar, Tripura 799022, India

## Abstract

Cancer is an inevitable matter of concern in the medicinal chemistry era. Chalcone is the well exploited scaffold in the anticancer domain. The molecular mechanism of chalcone at cellular level was explored in past decades. This mini review provides the most recent updates on anticancer potential of chalcones.

## 1. Introduction

**Figure 1:**
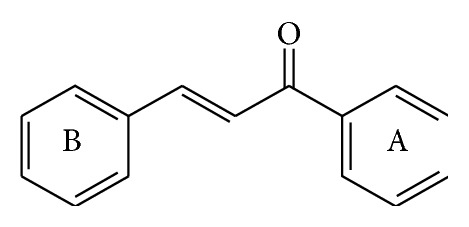
Chemical structure of chalcone.

Cancer, which is commonly a harbinger of imminent patient death, is a group of diseases characterized by uncontrolled growth and spread of abnormal cells and aberrant cell behavior, which leads to expansive masses that destroy surrounding normal tissue and is able to attack vital organs resulting in disseminated disease. It is an inevitable matter of concern in the medicinal chemistry era and an increasing burden to the population. According to estimates from the International Agency for Research on Cancer (IARC), the global burden is expected to grow to 21.4 million new cancer cases and 13.2 million cancer deaths by 2030. There were 12.7 million new cancer cases in 2008 worldwide, of which 5.6 million were in the economically developed countries and 7.1 million were in the economically developing countries. The corresponding estimates for total cancer deaths in 2008 were 7.6 million (about 21,000 cancer deaths a day), 2.8 million in the economically developed countries and 4.8 million in the economically developing countries [1, 2]. Pathophysiology of all cancers involves the malfunction of genes that control cell growth, division, and death. Cancers evolve through multiple changes resulting from a combination of hereditary and environmental factors, which mutate genes encoding critical cell-regulatory proteins. The new generations of anticancer drugs affect the signals that promote or regulate the cell cycle, growth factors and their receptors, signal transduction pathways, and pathways affecting DNA repair and apoptosis rather than targeting the direct synthesis of DNA. Chalcone derivatives of diverse chemical architectures are quite significant in anticancer drug discovery and hence are in the center of attention of drug hunters. Anticancer activity of chalcone might be due to molecular alteration such as induction of apoptosis, DNA and mitochondrial damage, inhibition of angiogenesis, tubulin inhibition, kinases inhibition, and also drug efflux protein activities. Chalcones are also implemented in cancer diagnosis too. Chalcone is chemically 1,3-diaryl-2-propen-1-one (Figure 1) in which the two aromatic rings are joined by a three-carbon *α*,*β*-unsaturated carbonyl system, representing a class of flavonoids that occur naturally in fruits and vegetables. Chalcones are also metabolic precursors of some flavonoids and isoflavonoids. The plants containing chalcone are traditionally employed for therapeutical purposes [3–7].

**Figure 2:**
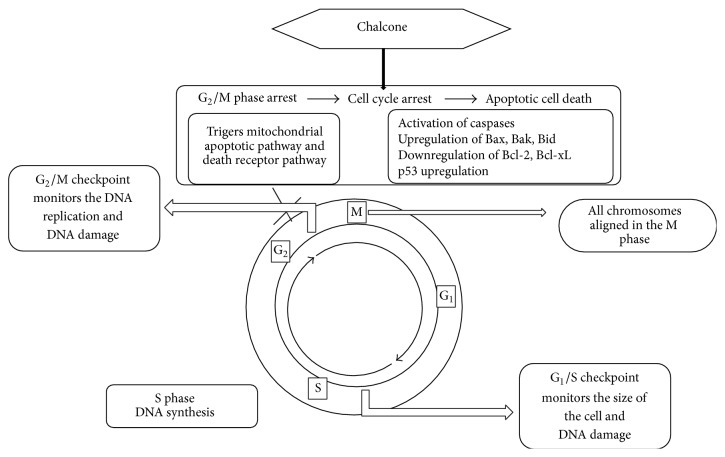
Action of chalcone on cell cycle.

Chalcones were exploited well for their wide application in pharmacological area. It is reported that chalcones have several advantages such as poor interaction with DNA and low risk of mutagenicity [8]. Hence, some clinically useful anticancer drugs have reported genotoxicity due to their interaction with amino groups in nucleic acids; chalcones may be devoid of these side effects due to their structural flexibility. Literature reveals that natural and synthetic chalcones are desirable to elicit cytotoxic and apoptotic activity (Figure 2). For this reason, chalcones have been well documented. They have been reviewed not only in the articles mentioned in the text but also in more recent articles, which have eluded the authors [9–11]. This review (surveyed database, 2013–2015) is complementary to the researchers for optimizing the lead chalcone as potential anticancer scaffold with increased potency.

## 2. Molecular Mechanism of Apoptosis

Cell death in tumors is a passive degradative reaction known as necrosis, most likely due to inadequate angiogenesis within the tumor cell. On the other hand, apoptotic cell death includes a number of gene families, and altering proapoptotic pathways specifically in tumors is something of a holy grail for oncology and medicinal chemistry. When cell production rate exceeds the cell loss rate through mitosis, it results in net tumor. Apoptosis is often referred to as an altruistic cell suicide process, where damaged DNA in the cell produces signals, resulting in both repair and apoptotic pathways, and if repair cannot be done then the cell undergoes apoptosis in a manner like “better dead than wrong.” The cells which harbour mutant p53 will have a survival advantage over normal cells. In response to DNA damage, normal cells upregulate p53 which acts as a transcription factor for cell cycle arrest and apoptosis; thereafter, p53-mutant cells become unable to carry out this protective arrest or apoptosis and might survive with what otherwise would turn to lethal genetic damage, perhaps explaining why p53 mutations are so common in human cancers. The decision of apoptosis is largely played out on the mitochondrial surface between three major families: the so-called three horsemen of apoptosis. The final executioner proteases called caspases play pivotal role by cleaving critical substrates such as DNA repair enzymes and cytoskeletal proteins, but they are stored as zymogens bound to an apoptotic adenosine triphosphate, apoptosis-activating factor-1 (Apaf-1), which is the mammalian homologue of the nematode* Caenorhabditis elegans* cell death protein, Ced-4. Once cytochrome c is released, it binds to the cytosolic protein Apaf-1 to facilitate the formation of apoptosome, which in turn activates apoptotic caspases. However, proapoptotic Bcl-2 family proteins such as Bax which is upregulated by p53 can activate apoptosis by releasing cytochrome c (cyt-c) from mitochondria, where Apaf-1 activation takes place [2, 6, 12, 13].

## 3. Molecular Insights of Anticancer Chalcones

Literature on anticancer chalcones highlights the employment of three pronged strategies, namely, structural manipulation of both aryl rings, replacement of aryl rings with heteroaryl scaffolds, and molecular hybridization through conjugation with other pharmacologically interesting scaffolds for enhancement of anticancer properties. Various substitutions on both aryl rings (A and B) of the chalcones, depending upon their positions in the aryl rings, appear to influence anticancer activity by interfering with various biological targets. Similarly, heterocyclic rings, either as ring A or as ring B in chalcones, also influence the anticancer activity shown by this class of compounds. Hybrid chalcones formulated by chemically linking chalcones to other prominent anticancer scaffolds such as benzodiazepines, benzothiazoles, and imidazolones have demonstrated synergistic or additive pharmacological activities too [6].

### 3.1. Chalcones against Leukemia Cell Lines

**Figure 3:**
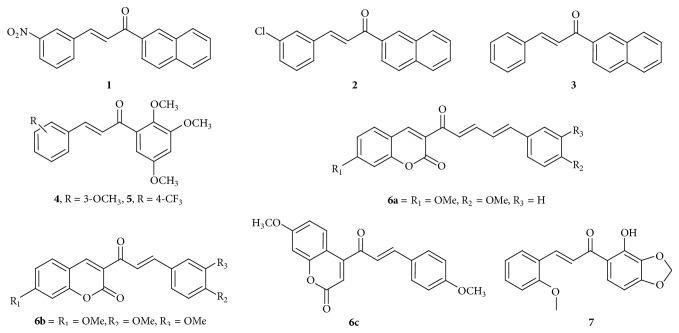
Chalcones against leukemia cell lines. (**1**) CC_50_ of 30 *μ*M, (**2**) CC_50_ of 40 *μ*M, and (**3**) CC_50_ of 40 *μ*M: increases proapoptotic proteins Bax, Bid, and Bak (only chalcone 2) and decreases antiapoptotic Bcl-2 expression. Caspase-9 and caspase-12 activation increases CHOP expression resulting in induction of ER stress. (**4**) IC_50_ values (4 to 8 *μ*M) and (**5**) IC_50_ values (4 to 8 *μ*M): induction of apoptosis through the reduction of the mitochondrial membrane potential, reduction in Bcl-2 expression, increase in Bax expression, and increase in active caspase-3. ((**6a**) to (**6c**)) IC_50s_ between 12 and 85 *μ*M for HDAC isoenzymes, inhibiting total HDAC activity by 20–50% at 100 *μ*M: histone deacetylases (HDACs) inhibitor. (**7**) IC_50_ of 13.7 ± 0.8 *μ*M: caspase-3 and caspase-7 activation.

Anticancer activity of three naphthyl chalcones,** 1**,** 2**, and** 3**, shown in Figure 3 was investigated by Winter et al. (2010) [14]. In a subsequent study, these chalcones were found to possess concentration- and time-dependent cytotoxicity on murine lymphoblastic leukemia cell line (L1210). Chalcones** 1**,** 2**, and** 3** induced apoptosis via an activated caspases-dependent pathway. The activities of caspase-8, caspase-9, and caspase-12 were found to increase after the treatment of L1210 cells with the CC_50_ of 30 *μ*M of chalcone** 1** and 40 *μ*M of chalcones** 2** and** 3**. It was found that chalcones** 1** and** 2** induced an increase of proapoptotic proteins Bax, Bid, and Bak (only chalcone** 2**), as well as a decrease in antiapoptotic Bcl-2 expression. These chalcones also induced an increase in death receptor Fas and a decrease in p21 and p53 expression. Chalcones** 1** and** 2** exert a statistically significant increase in cytochrome c release from mitochondria to cytoplasm. Cytochrome c release mediates caspase-9 activation, while the apoptosis inhibitors from Bcl-2 family prevent the efflux of cytochrome c from mitochondria. Chalcone** 3** seems to act by a different mechanism to promote cell death, as it did not change the mitochondrion-related proteins nor did it induce the cytochrome c release. Chalcones** 1**,** 2**, and** 3** induced an increase in cell calcium concentration and an increase in CHOP (C/EBP homologous protein) expression which is the transcription factor involved in endoplasmic reticulum stress, together with an increase in caspase-12 activity, suggesting that chalcones could induce an endoplasmic reticulum (ER) stress. They did not result in the arrest of G_0_/G_1_, S, or G_2_/M phases of the cell cycle in contrast to paclitaxel, which was used as a positive control in this study and induced a statistically significant enrichment of G_2_/M fraction. They presented a selectivity index (SI) higher than one, indicating that chalcones show higher selectivity to leukemic cells when compared to nontumoral cells (Vero = monkey kidney cells and NIH/3T3 = murine fibroblast). The only exception was chalcone** 3** which presented the same toxicity in L1210 and Vero cells. The selectivity index (SI) corresponds to CC_50_ (concentration of the compounds, which results in 50% of cell viability) of chalcones on nontumoral cell lines (Vero and NIH/3T3) divided by CC_50_ determined for cancer cells (L1210) [15]. 2′,4′,5′-trimethoxychalcones** 4**,** 5** (Figure 3) were found to reduce the viability of human K562 acute myeloid leukemia cell and human Jurkat acute lymphoid leukemia cell significantly and caused a cytotoxic effect in both cell lines in a concentration- and time-dependent manner. They were found to have low IC_50_ values (4 to 8 *μ*M) against both cell lines and did not have a cytotoxic effect on normal human lymphocytes. The mechanism of action involves the reduction of cell proliferation which has been shown by a reduction in the expression of cell proliferation marker Ki67. Cell death induced by 2′,4′,5′-trimethoxychalcones was due to induction of apoptosis through the reduction of the mitochondrial membrane potential, reduction in Bcl-2 expression, increase in Bax expression, increase in active caspase-3, and activating the intrinsic pathway and execution phase of apoptosis [16].

Histone deacetylases (HDACs) are enzymes which catalyze removal of acetyl groups from lysine residues. HDACs target nonhistone proteins including *α*-tubulin, heat shock protein 90, or p53. HDACs play a critical role in epigenetic gene regulation and hence control multiple cellular events. Hence, HDACs expressions and/or activity are deregulated in various cancer subtypes, leading to a target for anticancer therapy. Coumarin-containing compounds** 6a**,** 6b**, and** 6c** (Figure 3) were found to inhibit total HDAC activity by 20–50% at 100 *μ*M in chronic myeloid leukemia K562 and histiocytic lymphoma U937 cell lines. The activity of the compounds was compared to peripheral blood mononuclear cells (PBMCs) of healthy donors, which showed no effect on PBMC viability. In comparison to** 6a**, compound** 6b**, with a methoxy group at R_3_, presented increased levels of inhibition. Activity is due to methoxy group in the architecture. Compound** 6b** was tested against seven HDAC isoenzymes (1, 2, 3, 6, 8, 10, and 11) and acted as a pan-HDACi with IC_50s_ between 12 and 85 *μ*M.** 6b** inhibited HDAC3 with an IC_50_ at 12 *μ*M and may serve as a lead for targeting this nuclear isoenzyme [17]. Chalcone** 7** (Figure 3) with monomethoxy group at the ortho position showed a significant effect on downregulation of cancer cell proliferation and viability in three different leukemia cell lines (K562, Jurkat, and U937). The executioner caspase cleavage analyses indicated that the cytotoxic effect mediated by chalcone** 6** is due to induction of apoptotic cell death by caspase-3 and caspase-7 activation. The cytotoxic effect was cell type-specific and targeted preferentially cancer cells as peripheral blood mononuclear cells (PBMCs) from healthy donors were less affected by the treatment compared to K562, Jurkat, and U937 leukemia cells. It was observed that compound** 6** obeyed Lipinski's rule of five which indicates drug-likeness property of that molecule [18].

### 3.2. Chalcones against Colon Cancer Cell Lines

**Figure 4:**
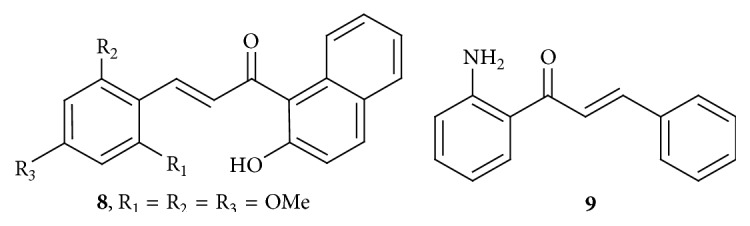
Chalcones against colon cancer cell lines. (**8**) IC_50_ of 14.5 ± 1.1 *μ*M: increases proapoptotic proteins Bax, Bid, and Bak (only chalcone 2) and decreases antiapoptotic Bcl-2 expression. Caspase 9 and caspase 12 activation increases CHOP expression resulting in induction of ER stress. (**9**) IC_50_ of 4.39 *μ*M: increases the death receptors expression (TRAIL-R1 and TRAIL-R2) (Tumor necrosis factor- (TNF-) related apoptosis-inducing ligand) and proapoptotic markers (p21, Bad, Bim, Bid, Bax, Smac, caspase-3, and caspase-8) as well as reducing the antiapoptotic markers (livin, XIAP, and HSP27).

2′-Hydroxy-2,4,6-trimethoxy-5′,6′-naphthochalcone,** 8** (Figure 4), had most effectively inhibited the clonogenicity of SW620 colon cancer cells. The IC_50_ value for compound** 8** was 14.5 ± 1.1 *μ*M against SW620 cells. Mechanistically, compound** 8** triggered cell cycle arrest at G_2_/M phase, followed by an increase in apoptotic cell death, which involves the disruption of microtubular networks. The mitotic spindle network is essential for appropriate segregation of chromosomes between the two daughter cells at cell division, where disruption leads to apoptosis. Compound** 8** was found to destabilize microtubule assembly during mitosis, associated with failure of mitotic spindle formation, in turn blocking mitosis at the prometaphase or metaphase-anaphase transition. The molecular mechanism is via both p53-dependent and caspase-2-mediated apoptosis. The DNA damage response has been found to be associated with** 8** induced apoptosis. However, the study did not distinguish between the DNA damage checkpoint and mitotic checkpoint pathways of apoptosis. The microtubule stabilizer paclitaxel and the destabilizer colchicine were used as reference compounds [19]. TRAIL (tumor necrosis factor- (TNF-) related apoptosis-inducing ligand) is a member of the TNF superfamily of cytokines. TNF family members contain highly conserved carboxyl-terminal domains and induce receptor trimerization for the transduction of intracellular signal. TRAIL is able to induce apoptotic cell death through caspase-dependent mechanisms. TRAIL binds to four different receptors, two of which are death receptors 4 and 5 (DR4 and DR5, resp.), induces apoptosis, and decoys receptors, DcR1 and DcR2, which lack the cytoplasmic death domain for transducing apoptotic death signals and protect cells from TRAIL induced cell death by interfering with signaling through DR4 and DR5.

It is generally believed that transformed tumor cells are more susceptible to TRAIL-mediated cell death owing to the selective loss of decoy receptors. Chalcone** 9** (Figure 4) (IC_50_ = 4.39 *μ*M) containing an amino (-NH_2_) group was the most potent and selective against TRAIL-resistant human colon cancer (HT-29) and nontoxic against normal human embryonic kidney (HEK-293) cells. 5-Fluorouracil was used as reference. It was observed that a large portion of HT-29 cells had undergone late apoptosis and/or necrosis after treatment with 5-fluorouracil (5 *μ*M). These findings were consistent to show that chalcone** 33** induces apoptosis rather than necrosis in HT-29 cells. It increases the death receptors expression (TRAIL-R1 and TRAIL-R2) and proapoptotic markers (p21, Bad, Bim, Bid, Bax, Smac, caspase-3, and caspase-8) as well as reducing the antiapoptotic markers (livin, XIAP, and HSP27) [20].

### 3.3. Chalcones against Cervix Adenocarcinoma Cell Lines

**Figure 5:**
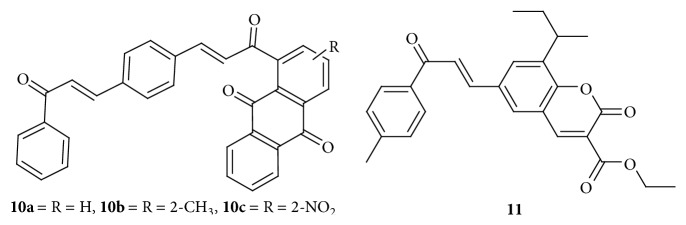
Chalcones against cervix adenocarcinoma cell lines. ((**10a**) to (**10c**)) IC_50_ values ranging from 2.36 to 2.73 *μ*M: increases p18 Bax, accumulation of cells in S, and G_2_/M phases. (**11**) IC_50_ of 4.7 to 7.6 *μ*M: induction of caspase-dependent intrinsic pathway p53 and its transcriptional target PUMA upregulation.

Anthraquinone based chalcone compounds were synthesized and evaluated for their anticancer potential, where compounds** 10a**,** 10b**, and** 10c** (Figure 5) showed promising activity in inhibition of cervix adenocarcinoma (HeLa) cells with IC_50_ values ranging from 2.36 to 2.73 *μ*M and low cytotoxicity against healthy human fetal lung fibroblast cell lines (MRC-5). Cisplatin (IC_50_ = 2.10 *μ*M) was taken as a reference. All the compounds cause the accumulation of cells in S and G_2_/M phases in a dose-dependent manner and induce caspase-3-dependent apoptosis and noncovalent interaction with the minor groove of the double-helical CT-DNA and the damage of DNA double helix structure. Significant increase of p18 Bax in treated HeLa cell line yielded consistency with the higher cytotoxic efficacy of compounds which implies the involvement of mitochondria in apoptotic process [21].

Synthesis and antiproliferative activity of a series of novel coumarin-chalcone hybrids were done by Singh et al. (2014). The most potent molecule of the series was compound** 11** (Figure 5). Regression of tumors induced by HeLa cell xenografts in* nod* SCID mice was found after oral administration of this molecule. It inhibited proliferation of cervical cancer cells (HeLa and C33A) by inducing apoptosis and arresting cell cycle at G_2_/M phase. Apoptosis was due to induction of caspase-dependent intrinsic pathway and alterations in the cellular levels of Bcl-2 family proteins. The mitochondrial transmembrane potential also got highly depleted in compound** 11** treated cells due to an increase in Bax/Bcl-2 ratio and intracellular ROS. Compound** 11** induced release of cytochrome c into the cytosol and activation of initiator caspase-9 and executioner caspase-3 and caspase-7. Tumor suppressor protein p53 and its transcriptional target PUMA (p53 upregulated modulator of apoptosis) were upregulated, suggesting their role in mediating the cell death. Based on dose response curves, the calculated IC_50_ (after 48 h treatment) was 4.7 ± 1.0 *μ*M for C33A cells and 7.6 ± 2.0 *μ*M for HeLa cells. Similar results were found in colony formation assay, where significant decrease in cellular colonies was observed in compound treated groups. Chalcone** 11** was apparently nontoxic to the animals as they did not show any loss of weight during experiment. Fluorescence microscopy revealed condensation and fragmentation of nuclei in the treated cells. Cell population with fragmented nuclei increased in a time-dependent manner with the addition of chalcone** 11**. Significant increase in apoptotic cells with fragmented nuclei, which is consistent with the changes in nuclear morphology and flow cytometry analysis, indicated induction of apoptosis by chalcone** 11** [22].

### 3.4. Chalcones against Lung and Breast Cancer Cell Lines

**Figure 6:**
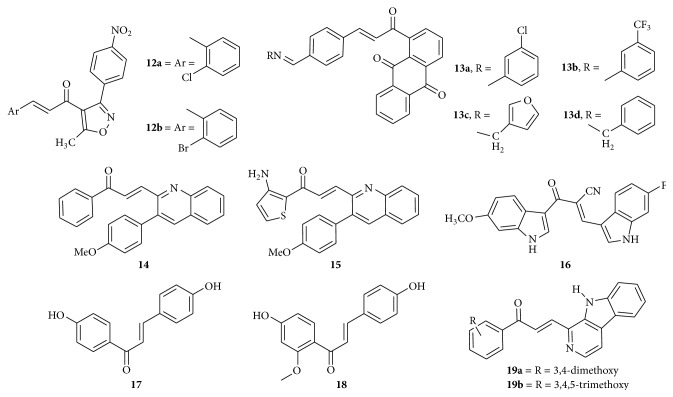
Chalcones against lung cancer and breast cancer cell lines. (**12a**) IC_50_ values of 1.35–2.07 *μ*M and (**12b**) IC_50_ values of 7.27–11.07 *μ*M: upregulation of the DR5; extrinsic pathway of apoptosis. ((**13a**) to (**13d**)) IC_50_ values ranging from 1.76 to 6.11 *μ*M: inhibited tubulogenesis, caspase-3 and caspase-8 activation, and inhibitory potential against matrix metalloproteinases (MMP-2) secretion. (**14**) IC_50_ values of 1.41 and 0.70 *μ*M: cell cycle arrest at G_2_/M phase, activation of caspase-3, and cleavage of PARP. (**15**) IC_50_ value of less than 0.10 *μ*M: cell cycle arrest at G_2_/M phase, activation of caspase-3, and cleavage of PARP. (**16**) IC_50_ of 0.8 *μ*M: enhances tubulin polymerization suggesting the activity as microtubule stabilizing agents. (**17**) IC_50_ of 0.16 to 0.44 *μ*M and (**18**) IC_50_ of 0.56 to 0.65 *μ*M: suppress the activation of NF-*κ*B pathway. (**19a**) IC_50_ of 2.25 *μ*M and (**19b**) IC_50_ of 3.29 *μ*M: induced DNA fragmentation and apoptosis.

Novel isoxazolyl chalcones were synthesized and evaluated for their activities* in vitro* against human non-small lung cancer cells (H1792, H157, A549, and Calu-1). Compounds** 12a** and** 12b** (Figure 6) were identified as the most potent anticancer agents with IC_50_ values ranging from 1.35 to 2.07 *μ*M and from 7.27 to 11.07 *μ*M against H175, A549, and Calu-1 cell lines, respectively. The structure-activity relationship study showed that compounds bearing electron withdrawing groups (EWG) at the 2-position of the phenyl ring in aryl group were more effective. Compounds** 12a** and** 12b** increased the percentage of cell in sub-G_0_ phase and decreased the percentage of cell in G_0_/G_1_ phase. These results suggested that** 12a** and** 12b** induced apoptosis in A549 cells in a dose-dependent manner. They have upregulated the DR5 in a dose-dependent manner which suggests the extrinsic pathways of apoptosis. Cleaved bands of caspase-8, caspase-9, caspase-3, and poly (ADP-ribose) polymerase (PARP) also indicated the induction of apoptosis [23]. Imine derivatives of hybrid chalcone analogues** 13a**,** 13b**,** 13c**, and** 13d** (Figure 6) containing anthraquinone scaffold were synthesized and evaluated for their* in vitro* cytotoxic activity against HeLa, LS174, and A549 cancer cells. Compound** 13c** with furan ring linked to imino group showed potent activity against all target cells with IC_50_ values ranging from 1.76 to 6.11 *μ*M, which are comparable to cisplatin. They inhibited tubulogenesis and compound** 13a** exhibited strong antiangiogenic effect. Generally, introduction of electron withdrawing substituents such as -Cl and -CF_3_ in metaposition of the phenyl ring was found to exert good cytotoxicity. Compounds did not have tendency to significantly accumulate the cells in sub-G_1_ phase, except compound** 13a**, which led to an increase in sub-G_1_ (observed only in cells undergoing apoptosis) phase in A549 cells as well as changes in G_2_/M arrest of A549 and LS174 cells. Compounds induced the activities of caspase-3 and caspase-8 in treated HeLa cells and exhibited strong antiangiogenic activity by showing potent inhibitory potential against matrix metalloproteinases (MMP-2) secretion. Strong implication of MMP-2 is found in angiogenesis and promotes tumor metastatic potential playing crucial role not only in invasion but also in determination of cancer cell transformation, growth, apoptosis, and signal transduction pathways [24]. 3-Phenylquinolinylchalcone derivatives,** 14** and** 15** (Figure 6), were synthesized and evaluated for their antiproliferative activities. Compound** 14** was active against the growth of non-small cell lung cancer cells H1299 and SKBR-3 breast cancer cells with IC_50_ values of 1.41 and 0.70 *μ*M, respectively, which was more active than the positive topotecan (IC_50_ values of 6.02 and 8.91 *μ*M, resp.). Compound** 15** exhibited an IC_50_ value of less than 0.10 *μ*M against the growth of MDA-MB231 in a dose- and time-dependent manner and was noncytotoxic to the normal mammary epithelial cell (H184B5F5/M10).

Mechanism studies indicated that compound** 15** induced cell cycle arrest at G_2_/M phase. The proportion of cells was decreased in G_1_ and accumulated in G_2_/M phase after 24 h treatment of compound** 15**, while the hypodiploid (sub-G_0_/G_1_ phase) cells increased. Compound** 15** caused activation of caspase-3, cleavage of PARP, and consequently the cell death. Caspase-3 is an executioner caspase whose activation leads to the cleavage of key cellular proteins including DNA repair enzyme poly (ADP-ribose) polymerase which is involved in DNA repair predominantly in response to environmental stress and is important for the maintenance of cell viability [25]. *α*-Cyano bis(indolyl)chalcone** 16** (Figure 6) was found to be the most potent and selective against A549 lung cancer cell line (IC_50_ = 0.8 *μ*M)* in vitro* which is time-dependent. The activity is due to the presence of methoxy and fluoro substituents on indole rings. It was found to enhance tubulin polymerization suggesting the activity as microtubule stabilizing agents, though it was a preliminary study [26]. NF-*κ*B pathway has been shown to be critical to survival of lung cancer cells, and many natural products obtained from plants were found to inhibit the activation of this pathway. Two cardamonin analogs, 4,4′-dihydroxylchalcone,** 17**, and 4,4′-dihydroxy-2′-methoxychalcone,** 18** (Figure 6), were found to suppress the activation of NF-*κ*B pathway in lung cancer cells. They were also shown to suppress the growth of A549 xenograft mice model. Compound** 17** inhibited the survival of primary lung cancer cells with IC_50_ values of 0.44 ± 0.05 and 0.16 ± 0.03 *μ*M, respectively, against two lung cancer cells tested. Compound** 18** inhibited their survival with similar IC_50_ values (0.56 ± 0.12 *μ*M and 0.65 ± 1.36 *μ*M). Immunoblot analysis displayed that caspase-3 and PARP were both cleaved in the two lung cancer cell lines treated with compounds** 17** and** 18**, indicating the activation of apoptotic pathway. Subsequent estimation of the phosphorylation level of* IKKβ*, NF-*κ*B pathway stimulating kinase, by immunoblotting assay demonstrated that both decreased* IKKβ* phosphorylation in lung cancer cell lines including A549, NCI-H460, and NCI-H1668 and lead to the accumulation of* IκBα* and* IκBβ* and the export of nuclear p65 transcription factor. From the immunofluorescent staining, it was shown that transcription factor p65, which is a downstream effect of NF-*κ*B pathway, was sequestered in the cytoplasm of A549 cells treated by compound** 17** or compound** 18**. Incorporation of luciferase reporter system also results in transcription activity of NF-*κ*B in lung cancer cells treated with compound** 17** or compound** 18** and demonstrated that relative luciferase expression was significantly reduced in compound** 17** or compound** 18** treated lung cancer cells. In addition, the transcription of target genes of NF-*κ*B pathway, Bcl-2, and survivin was found to be suppressed in lung cancer cells treated with the two compounds. Compounds** 17** and** 18** suppressed the growth of lung cancer xenograft in nude mice model. Higher concentration of the two compounds greatly inhibited the growth of tumors by 71.0 and 79.1%, respectively, although inhibitory effect was not significant at lower concentration. Compared with compound** 17**, compound** 18** appeared to be slightly more potent to retard the growth of established tumors. Methoxy substitution is found to be effective [27]. *β*-Carboline based chalcones were synthesized and evaluated for their cytotoxic activity against a panel of human cancer cell lines. Compounds** 19a** and** 19b** (Figure 6) showed very good antiproliferative activity against breast cancer cell line (MCF-7), where IC_50_ values were recorded at 2.25 *μ*M and 3.29 *μ*M, respectively. Furthermore, compound** 19a** markedly induced DNA fragmentation and apoptosis in breast cancer cells, which is an essential step in the cells undergoing apoptosis [28].

### 3.5. Chalcones against Hepatocellular Carcinoma Cell Lines

**Figure 7:**
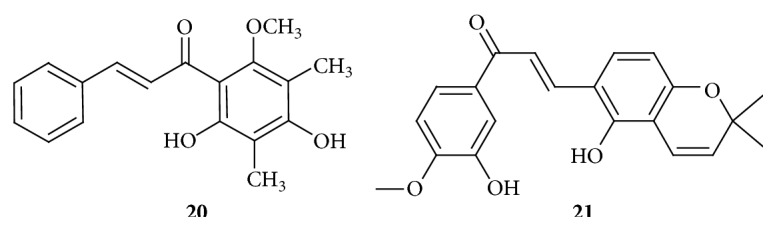
Chalcones against hepatocellular carcinoma cell lines. (**20**) IC_50_ of 6.25  *μ*M: mitochondria dependent pathway involving inhibition of Bcl-2 expression leading to disintegration of the outer mitochondrial membrane. Caspase-3 and caspase-9 activities. (**21**) IC_50_ of 10 *μ*M: hypoxia inducible factor-1 (HIF-1) inhibitor.

The molecular mechanism to induce apoptosis in human hepatoma SMMC-7721 cells was investigated by using 2′,4′-dihydroxy-6′-methoxy-3′,5′-dimethylchalcone,** 20** (Figure 7), isolated from the buds of* Cleistocalyx operculatus*. It inhibits proliferation of SMMC-7721 cells by inducing apoptosis. Compound** 20** induced apoptosis via a mitochondria dependent pathway involving inhibition of Bcl-2 expression leading to disintegration of the outer mitochondrial membrane. Further downstream of the apoptosis cascade, compound** 20**, increases caspase-3 and caspase-9 activities [29].

Angiogenesis and invasion are two highly interconnected events in cancerous cells where involvement of multiple molecules and pathways takes place. Hypoxia inducible factor-1 (HIF-1) has been identified as an important protein involved in those events. Activated HIF-1 controls the expression of genes involved in tumor cell immortalization, stem cell maintenance, metabolic reprogramming, autocrine growth, and treatment failure, thereby making it an important target for the development of new antitumor therapy. A novel series of chalcone derivatives were synthesized and their biological activities against HIF-1 were evaluated by Wang et al. (2015). It has been found that compound** 21** (Figure 7) exhibited inhibitory effects on HIF-1 at 10 *μ*M concentration by downregulating the expression of HIF-1*α* and HIF-1*β* under hypoxic conditions. The HIF inhibition rate has been found to be 83.0 ± 16.9%, while topotecan, HIF-1*α* inhibitor, was used as control and for comparison it has the inhibition rate of 82.8 ± 2.9%. In addition, compound** 21** displayed inhibitive effect against the migration and the invasion of Hep3B (human liver cancer cell line) and human umbilical vein endothelial cells (HUVEC) and tube formation of HUVECs and significant antiangiogenic and anti-invasive activities. From the* in vivo* study it has been found that** 21** could retard tumor growth of Hep3B xenograft models and reduced CD31 and MMP-2 expression in tumor tissues. CD31 (cluster of differentiation 31) is platelet endothelial cell adhesion molecule (PECAM-1), which is a protein found to be expressed in certain tumors. Compound** 27** was well tolerated and was found to be nontoxic up to 200 mg/kg in Swiss mice in acute intravenous toxicity [30].

### 3.6. Chalcones against Bladder and Prostate Cancer Cell Lines

**Figure 8:**
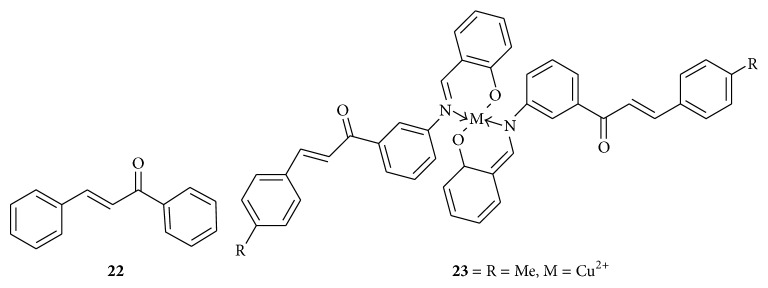
Chalcones against bladder and prostate cancer cell lines. (**22**) Concentration of 6 *μ*g/mL: reduction in the expression of cyclin A and cyclin B1, decrease in the expression of Cdc2 and an increase in the expression of p21 and p27, increase in the expression of proapoptotic proteins Bax and Bak, and decrease in the expression of antiapoptotic proteins Bcl-2, Bcl-xL proteins; NF-*κ*B activation by increasing the expression of I*κ*B*α* in cytoplasm leads to apoptosis. (**23**) IC_50_ value of 5.95 *μ*M: copper ions might act as penetration enhancers of chalcones into cancer cells and might act as inhibitors of drug efflux proteins.

The molecular mechanisms of growth inhibition in human bladder cancer cell lines T24 and HT-1376 by chalcone** 22** (Figure 8) are due to the induction of apoptosis, cell cycle progression inhibition by regulating cell cycle related factor like significant reduction in the expression of cyclin A and cyclin B1, decreases in the expression of Cdc2, and also increases in the expression of p21 and p27.

So, chalcone may cause cell cycle arrest by reducing the complex of Cdc2/cyclin B due to downregulation of multiple G2/M regulating proteins. It triggers the mitochondrial apoptotic pathway by releasing cytochrome c from the cytoplasm with an increase in caspase-9 and activating caspase-3. It is also increases the expression of proapoptotic proteins Bax and Bak, which are responsible for the initiation of mitochondrial apoptotic pathway, and decreases the expression of antiapoptotic proteins Bcl-2 and Bcl-xL, while overexpression of Bcl-2 and Bcl-xL is associated with enhanced oncogenic potential. Chalcone inhibits the NF-*κ*B activation by increasing the expression of I*κ*B*α* in cytoplasm, leading to apoptosis [31]. The copper chelate complex** 23** (Figure 8) showed promising activity with IC_50_ value of 5.95 *μ*M against PC3 cancer cell line* in vitro*. The estimated IC_50_ value for doxorubicin was 8.7 *μ*M, which was used as positive control. It was found that cancer cells have high affinity to copper ions; thus, upon chelation of chalcones with copper they are thought to be more liable to target cancer cells in microdendron architecture. It was assumed that copper ions might act as penetration enhancers of chalcones into cancer cells. Even if copper ions are noncytotoxic to cancer cells, they might act as inhibitors of drug efflux proteins characteristic to cancer cells. Drug efflux proteins, such as P-glycoproteins (Pgp), breast cancer resistance protein (BCRP), and multidrug resistance-associated protein 1 (MRP1), are responsible for drug resistance of cancer cells. The high cytotoxic activity is attributed to the presence of the p-methylphenyl moiety of ring B and the copper ion [32].

### 3.7. Chalcones against Multiple Cancer Cell Lines

**Figure 9:**
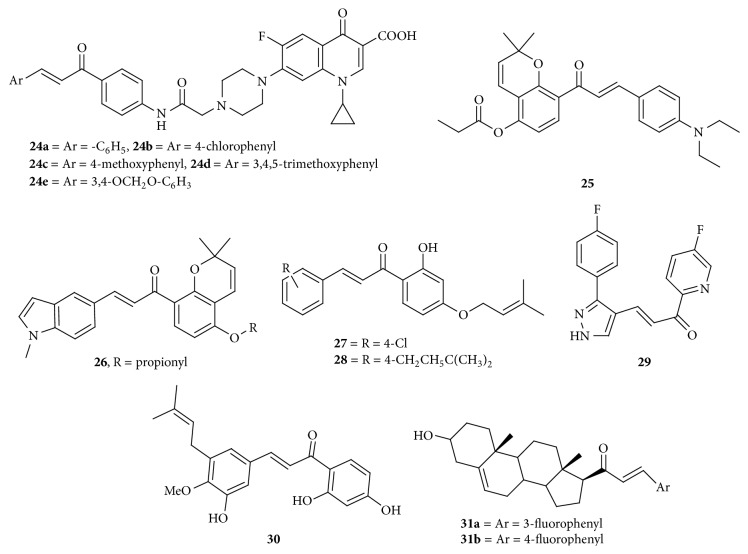
Chalcones against multiple cancer cell lines. ((**24a**) to (**24c**)) GI_50_ of 0.21 to 57.6 *μ*M: topoisomerase-I and topoisomerase-II inhibition. (**25**) IC_50_ of 0.15 to 0.52 *μ*M: arrest cells in the G_2_/M phase of the cell cycle and inhibit the polymerization of tubulin. Binds into the colchicine binding site of tubulin (molecular docking study). (**26**) IC_50_ values of 0.22 to 1.80 *μ*M: induced cell cycle arrest in G_2_/M phase and inhibited the polymerization of tubulin; binds at the colchicine binding site of tubulin (from molecular docking analysis). (**27**) IC_50_ value of 0.89 *μ*mol/L: cell division cycle 25 (CDC25) inhibitor. (**28**) IC_50_ values of 1.76 *μ*mol/L: cell division cycle 25 (CDC25) inhibitor. (**29**) IC_50_ value of 0.01 *μ*g/mL and 0.04 *μ*g/mL: molecular mechanism not studied. (**30**) IC_50_ values of 6.4 *μ*M and 8.5 *μ*M: cytotoxic, molecular mechanism not studied. (**31a**) IC_50_ value of 1.02 *μ*M and 0.79 *μ*M and (**31b**) IC_50_ value of 0.81 *μ*M and 1 *μ*M: cytotoxic.

Hybrids of N-4-piperazinyl-ciprofloxacin-chalcone were prepared by Abdel-Aziz et al. (2013). National Cancer Institute (NCI) selected 5 compounds, namely,** 24a**,** 24b**,** 24c**,** 24d**, and** 24e** (Figure 9), to carry out* in vitro* one-dose anticancer assay against full NCI 60 cell lines derived from leukemia, lungs, colon, renal, melanoma, CNS, prostate, ovarian, and breast cancer cell lines. Of the selected compounds,** 24a** and** 24d** exhibited the highest ability to inhibit the proliferation of different cancer cell lines while undergoing one-dose anticancer test. Among the nine tumor subpanels tested for, compound** 24a** was found to have broad-spectrum antitumor activity with selectivity ratios ranging between 0.42 and 1.87 at the GI_50_ level without producing selectivity, while compound** 24d** showed high selectivity towards the leukemia subpanel with selectivity ratio of 6.71 at GI_50_ level. Moreover compound** 24d** showed remarkable anticancer activity against most of the tested cell lines with GI_50_ ranging from 0.21 to 57.6 *μ*M. Furthermore, compounds** 24c** and** 24e** have shown remarkable topo-II inhibitory activity compared to etoposide at 100 *μ*M and 20 *μ*M concentrations. Compounds** 24c** and** 24e** were found to be potent topo-I inhibitors at 20 *μ*M concentration when compared to camptothecin. The results that were obtained indicated that introduction of N-4-piperazinyl moiety of ciprofloxacin into the chalcones derivatives dramatically increases the anticancer activity of the tested compounds severalfold higher than that of the activity shown by weak anticancer ciprofloxacin. Increase in antiproliferative activity of the tested compounds might be the result of either synergistic effect of the chalcone derivatives and/or alteration of the physicochemical properties of ciprofloxacin. To determine the DNA unwinding properties of** 24c** and** 24e**, amsacrine (m-AMSA), a well-known DNA intercalator, was chosen as a standard where a supercoiled pHOT1 DNA was used as a substrate since unwinding of the double strand of DNA helix is a practical characteristic of intercalating drugs. Amsacrine blocked unwinding of pHOT1 DNA in the presence of excess topo-I in dose-dependent manner, while compounds** 24c** and** 24e** did not block DNA unwinding at high concentration up to 1000 *μ*M treatment. The assay indicated that** 24c** and** 24e** do not interact with DNA but interact with topoisomerases for their inhibitory activity [33]. Compound** 25** (Figure 9) with a propionyloxy group at the 4-position of the left phenyl ring showed the most potent inhibition activity which inhibited the growth of HepG2, A549, A375, SMMC-7221, and K562 cancer cell lines with IC_50_ values of 0.15, 0.36, 0.62, 0.61, and 0.52 *μ*M, respectively. Compound** 25** was also shown to arrest cells in the G_2_/M phase of the cell cycle and inhibit the polymerization of tubulin. Molecular docking study suggested that compound** 25** binds into the colchicine binding site of tubulin. In summary, these findings suggest that compound** 23** is a promising new antimitotic compound for the potential treatment of cancer [34]. A new series of pyrano chalcone derivatives containing indole moiety synthesized by Wang et al. (2014) were evaluated for antiproliferative activities. Among those, compound** 26** (Figure 9), with a propionyloxy group at the 4-position of the phenyl ring A and N-methyl-5-indolyl on the B ring displayed the most potent cytotoxic activity against all tested cancer cell lines including multidrug resistant phenotype, which inhibits cancer cell growth with IC_50_ values ranging from 0.22 to 1.80 *μ*M. The tested cell lines were SMMC-7221, HepG2 (liver), PC-3 (prostate), A549 (lung), K562 (leukemia), HCT116 (colon), SKOV3 (ovarian), MCF-7 (breast), vincristine resistant HCT-8 (HCT-8/V), and taxol resistant HCT-8 (HCT-8/T). Compound** 26** was the most potent towards HepG2 cells with IC_50_ value of 0.22 *μ*M. Colchicine (2.5 *μ*M) and paclitaxel (2.5 *μ*M) were used as a reference. Compound** 26** produced cytotoxic activity in normal human liver cell line (LO2) above the concentration of 10 *μ*M. It significantly induced cell cycle arrest in G_2_/M phase and inhibited the polymerization of tubulin. It was also shown from molecular docking analysis that compound** 26** binds at the colchicine binding site of tubulin. Chalcone** 26** also exerted potent anticancer activity in HepG2 xenografts in BALB/c nude mice, where the growth of tumors in mice treated with chalcone** 26** (30 mg/kg) was reduced by 56.58% compared with that in control mice treated with vehicle only at day 35. Positive control reduced the growth of tumors by 25.93% [35].

**Figure 10:**
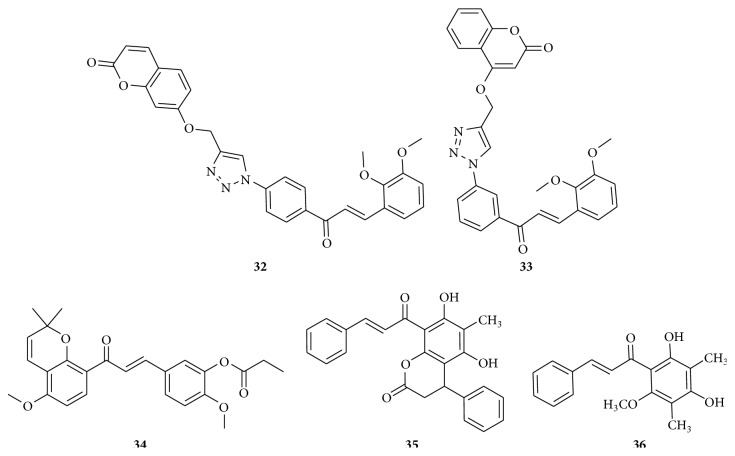
Chalcones against multiple cancer cell lines. (**32**) IC_50_ = 0.53 *μ*M and (**33**) IC_50_ value of 8.18 *μ*M: could snugly occupy the colchicine binding site of *β*-tubulin (molecular docking study). (**34**) Concentration of 0.2 *μ*M: cell cycle arrest in G_2_/M phase promoted tubulin polymerization into microtubules and caused microtubule stabilization similar to paclitaxel. (**35**) IC_50_ values of 1.60 *μ*M and (**36**) IC_50_ values of 2.82 *μ*M: inhibition of clonogenicity.

Cell division cycle 25 (CDC25), which includes CDC25A, B, and C homologues, a subfamily of dual-specificity protein tyrosine phosphatases, plays a role in the regulation of the cell cycle. The CDC25A and CDC25B isoforms are overexpressed in primary tissue samples from various human cancers, which is strongly associated with tumor aggressiveness and poor prognosis. CDC25B controls cell cycle progression by catalyzing removal of covalently attached contiguous phosphates and the subsequent activation of cyclin-dependent kinase 1 (Cdk1). CDC25B has been proposed to regulate reentry into mitosis after DNA damage. CDC25B overexpression has been documented in a variety of human cancers, including head and neck and colon, and non-small cell lung cancer validates its oncogenic potential. A series of 2′-hydroxy-4′-isoprenyloxychalcone derivatives were evaluated for the inhibition of CDC25B activity by Zhang et al. (2014). Among those, two compounds,** 27** and** 28** (Figure 9), have been found to have the potent inhibition activity and significantly inhibited CDC25B with inhibition rates of 99.95% and 99.75%, respectively, at a dose of 20 *μ*g/mL, which is similar to the known tyrosine phosphatase inhibitor sodium orthovanadate.* In vitro* cytotoxic activity assays showed that compounds** 27** and** 28** were potent against HCT116, HeLa, and A549 cells. They were particularly cytotoxic against colon HCT116 cancer cell line with IC_50_ values of 0.89 and 1.76 *μ*mol/L, respectively, for compounds** 27** and** 28**. Irinotecan (IC_50_ = 2.14 *μ*mol/L), a clinically relevant camptothecin, was chosen as a reference drug. In colo205 xenograft BALB/c nude male mice, compound** 27** produced a tumor volume inhibition of about 50% [36]. A new series of pyrazole chalcones were screened for the* in vitro* anticancer activities against MCF-7 (human breast adenocarcinoma) and HeLa (human cervical tumor cells) cell lines. Compound** 29** (Figure 9) showed the highest inhibition in human MCF-7 and HeLa cell lines with an IC_50_ value of 0.018 *μ*g/mL and 0.047 *μ*g/mL, respectively. The IC_50_ for standard doxorubicin in HeLa and MCF-7 cells was 1.15 *μ*g/mL and 1.84 *μ*g/mL, respectively. The activity is due to the presence of 4-fluorophenyl and 5-fluoropyridin moieties [37]. New prenylated chalcone,** 30** (Figure 9), named as renifolin C, was isolated from whole* Desmodium renifolium* plants and cytotoxicity was evaluated against five human tumor cell lines (NB4, A549, SHSY5Y, PC3, and MCF7) using the MTT method, using paclitaxel as a positive control. The IC_50_ values were 6.4 *μ*M and 8.5 *μ*M against NB4 and PC3 cell lines and above 10 *μ*M for the other cell line tested [38]. Various benzylidene pregnenolones were synthesized and* in vitro* cytotoxicity studies were performed by Banday et al. (2014). Compounds** 31a** and** 31b** (Figure 9) were found to be active against the HCT-15 (colon) and MCF-7 (breast) cell lines. Chalcone** 31a** has IC_50_ value of 1.02 *μ*M and 0.79 *μ*M, respectively, against HCT-15 and MCF-7, while** 31b** has 0.81 *μ*M and 1 *μ*M, respectively. Electron withdrawing groups have an effect over the activity [39]. Chalcone-coumarin hybrid** 33** (Figure 10) (IC_50_ = 0.53 *μ*M) displayed cytotoxicity against human lymphoblastic leukemia cell line (MOLT-3), where etoposide (IC_50_ = 0.05 *μ*M) was taken as a standard. Compounds** 32** and** 33** shown in Figure 10 displayed higher cytotoxic potency against HepG2 cells than the control drug, etoposide. The hybrid** 33** (IC_50_ = 4.26 *μ*M) was potent but was found to be toxic toward noncancerous African green monkey kidney cell line (Vero). Though analog** 32** displayed cytotoxic activity against HepG2 with IC_50_ value of 8.18 *μ*M, it was found to be nontoxic against Vero cells. It has been shown from molecular docking study that compounds could snugly occupy the colchicine binding site of *β*-tubulin [40].

A series of millepachine derivatives were synthesized by Cao et al. (2013). Among them, potent chalcone** 34** (Figure 10) induced apoptotic cell death of a wide variety of tumor cell lines including multidrug resistant human tumor cell lines in the nanomolar range by attacking microtubules. Flow cytometric study showed that chalcone** 34** significantly induced cell cycle arrest in G_2_/M phase at 0.2 *μ*M concentration.* In vitro* immunofluorescence staining and tubulin polymerization assay displayed that chalcone** 34** promoted tubulin polymerization into microtubules and caused microtubule stabilization similar to paclitaxel, which is suggesting that chalcone** 34** is a microtubule stabilizer via enhancing the rate of tubulin polymerization. Then, in* in vivo* study,** 34** exhibited potent inhibitory activities in human HepG2 xenograft tumor models [41]. Chalcone derivatives, named parasiticins C,** 35** and** 36** (Figure 10), were isolated from the leaves of* Cyclosorus parasiticus*. The* in vitro* cytotoxic activities were evaluated against lung cancer A549, hepatocellular carcinoma HepG2, breast cancer MCF-7 and MDA-MB-231, leukemia ALL-SIL, and pancreatic cancer SW1990. Compounds** 35** and** 36** exhibited substantial cytotoxicity against all six cell lines, especially toward HepG2 with the IC_50_ values of 1.60 and 2.82 *μ*M, respectively, which is comparable to the doxorubicin positive control (1.19 *μ*M). They induced apoptosis in the HepG2 cell line. In the clonogenicity assay on HepG2 cell line, capacity of single cancer cells to generate colonies was almost completely blocked by compound** 35** at a concentration of 2.4 *μ*M. Compound** 35** showed more potent cytotoxicity than** 36**, suggesting that the presence of an additional lactone ring fused enhances the cytotoxic activity [42].

### 3.8. Chalcone obtained by Using a Fission Yeast Bioassay

A fission yeast,* Schizosaccharomyces pombe*, was utilized in bioassay guided screening on several plant extracts to search for anticancer agents by Sánchez-Picó et al. (2014).

**Figure 11:**
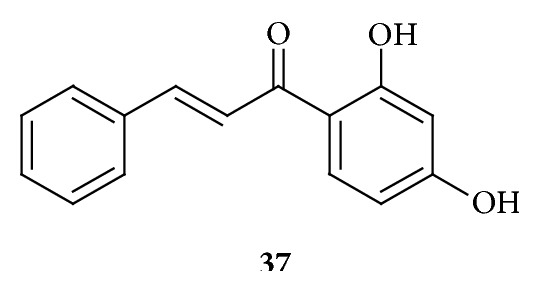
Chalcone obtained by using a fission yeast bioassay. (**37**) Concentration of 8 *μ*M to 20 *μ*M induces DNA damage which blocks cell cycle progression at the G_2_/M transition in a Rad3-dependent and Chk1-dependent manner.

It was found that an extract from leaves of* Corema album* belonging to family Ericaceae had antiproliferative activity. 2′,4′-Dihydroxychalcone,** 37** (Figure 11), the promising cytotoxic compound, was found in this extract and cytotoxic activity is likely due to its ability to induce DNA damage which blocks cell cycle progression at the G_2_/M transition in a concentration of 8 *μ*M to 20 *μ*M. Compound** 37** could induce DNA damage or interfere with DNA replication, which would eventually result in the activation of the cell cycle checkpoints, thus blocking cell cycle in G_2_. In subsequent study, deletions of Rad3, Chk1, and Cds1, three key kinases required for DNA checkpoints operating at G_2_/M, were done. Rad3 is required in order to signal downstream of either DNA replication problems or DNA damage, whereas Chk1 transmits DNA damage-induced signals and Cds1 signals in response to defective DNA replication. It was found that both Rad3 and Chk1 deletions suppressed the cell cycle block produced by compound** 37**, while Cds1 deletion showed partial suppression, suggesting that the G_2_/M block observed in compound** 37** is mostly caused by the activation of DNA damage checkpoint response. Methyl methanesulfonate (MMS), an alkylating compound that induces DNA damage, was used as control. Thus, compound** 37** (8–20 *μ*M) directly or indirectly causes damage to the DNA which results in a Rad3-dependent and Chk1-dependent cell cycle block at the G_2_/M transition [43].

### 3.9. Chalcone against Cathepsin B and Cathepsin H Obtained from Goat Liver

Cathepsins have emerged as a potential target for chemoprevention and anticancer therapy. Increased levels of cathepsin B and cathepsin H in a variety of tumors have shown their contribution towards invasion and metastasis.

**Figure 12:**
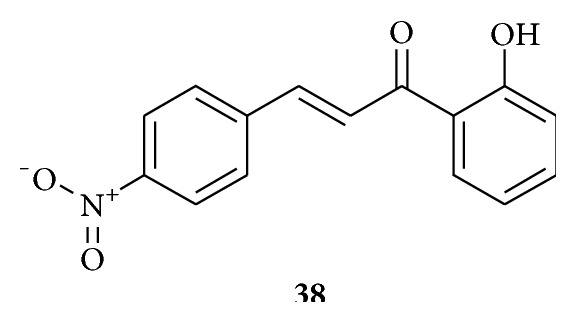
Chalcone against cathepsin B and cathepsin H obtained from goat liver. (**38**) *K*_*i*_ values of 6.18 × 10^−8^ M for cathepsin B. *K*_*i*_ values of 2.8 × 10^−7^ M for cathepsin H: cathepsin B and cathepsin H inhibitor.

Molecule** 38** (Figure 12) has been found to be potent among the synthesized inhibitors, which inhibits cathepsin B and H. The *K*_*i*_ (inhibition constant) value against cathepsin B was 6.18 × 10^−8^ M and 2.8 × 10^−7^ M for cathepsin H. The inhibition was of competitive type. 2′-Hydroxychalcones have been evaluated as potential inhibitors to these cysteine proteases probably due to presence of *α*,*β*-unsaturated carbonyl moiety in the molecule. -CH_2_SH moiety present at the active site of enzymes can interact with the electron deficient center present in the chalcones inhibiting the enzymes activity. Nitrosubstitution is useful for an effective interaction with cathepsins B and H. Its inhibition to cathepsin B as compared with cathepsin H indicates that cathepsin B's active site is more susceptible to these compounds as compared to cathepsin H. The results were also found to be consistent when compared with* in silico* docking studies [44].

### 3.10. Chalcones against Brain Cancer Cell Lines

**Figure 13:**
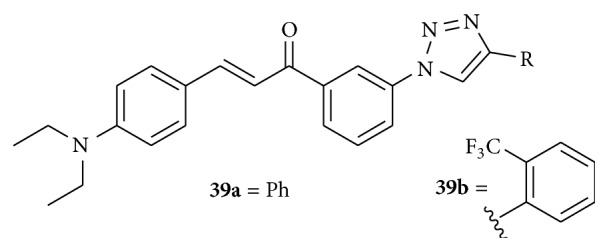
Chalcones against brain cancer cell line. ((**39a**) and (**39b**)) cotreatment of 10 *μ*M with TRAIL (25 ng/mL): TRAIL sensitizers.

Cotreatment with a chemosensitizer such as compounds** 39a** and** 39b** (Figure 13) and TRAIL was found to be more efficient to treat GBM (Glioblastoma Multiforme) than treatment with TRAIL alone.

GBM is one of the most aggressive forms of human malignant brain tumors, characterized by rapid growth, extensive invasiveness, and robust neoangiogenesis. Against human CRT-MG astroglioma cells by using the lactate dehydrogenase (LDH) release assay, it was found that compounds** 39a** and** 39b** with TRAIL (25 ng/mL) showed good cytotoxicity. 2-Ethylamino and substituted triazole groups are promising templates in the backbone of chalcone to develop TRAIL sensitizers as anticancer agents [45].

### 3.11. Chalcone against Multidrug Resistant Cancer Cell Lines

**Figure 14:**
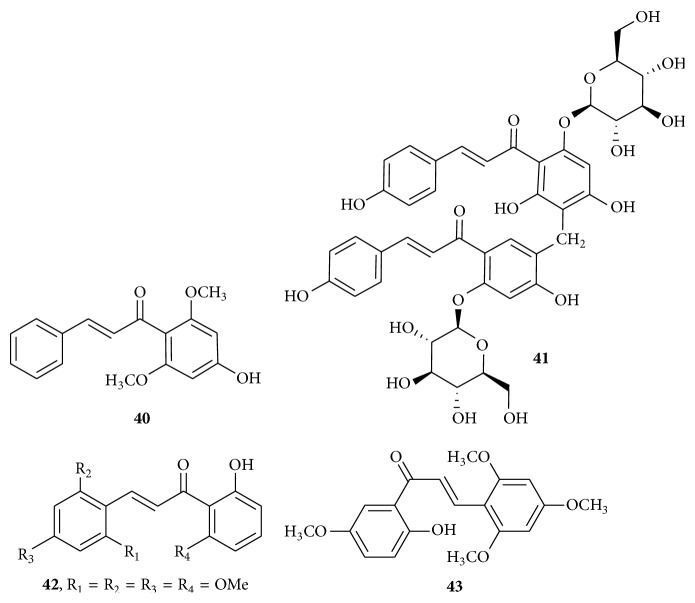
Chalcone against multidrug resistant cancer cell lines. (**40**) IC_50_ value of 2.54 *μ*M: arresting the cell cycle between G_0_/G_1_ phases, which is via the strong induction of apoptosis by disrupting mitochondrial membrane potential (MMP) and an increased production of reactive oxygen species (ROS). (**41**) Combined effects of** 36** and Tipifarnib reduce the IC_50_ value of Tipifarnib from 9.0 *μ*M (applied alone) to 3.8 *μ*M: increased the expression of HIF-1*α*. (**42**) GI_50_ of 3.87 *μ*M and (**43**) GI_50_ of 3.95 *μ*M: trigger cell cycle arrest at the G_2_/M phase and apoptotic cell death, aurora A kinase inhibitor.

Chemoresistance is a clinical problem that severely limits treatment success. MDR (multidrug resistance) can be defined as the ability of cancer cells to survive exposure to different chemotherapeutic agents and is a major cause of treatment failure in human cancers. 4′-Hydroxy-2,6′-dimethoxychalcone,** 40** (Figure 14), was isolated from* Polygonum limbatum* with IC_50_ values of 2.54 *μ*M against multidrug resistant CEM/ADR5000 leukemia cells. P-glycoprotein-expressing and multidrug resistant CEM/ADR5000 cells were much more sensitive toward compound** 40** than doxorubicin (IC_50_ = 195.12 *μ*M), which is a reference drug. Compound** 40** (Figure 14) has been found to arrest the cell cycle between G_0_/G_1_ phases, which is via the strong induction of apoptosis by disrupting mitochondrial membrane potential (MMP) and an increased production of reactive oxygen species (ROS) in the studied leukemia cell line. It was found that caspases were not involved in the apoptotic pathway induced by compound** 40**. The presence of hydroxyl (-OH) and methoxy (-OCH_3_) substituents influences the activity of compound** 40** [46].

The membrane transporter P-glycoprotein (P-gp), encoded by* mdr1* gene, is the main mechanism responsible for resulting in decreased intracellular drug accumulation in human MDR cancers. Mitogen activated protein (MAP) kinase pathway is involved in P-gp-mediated MDR. Dimer of chalcone named as tomoroside B,** 41** (Figure 14), was isolated from the aerial parts of* Helichrysum zivojinii*. The combined effects of chalcone dimer** 41** and Tipifarnib (the inhibitor of MAP kinase signaling cascade, a farnesyltransferase inhibitor) in MDR cancer cell line, NCI-H460/R, were found. These results point to the ability of compound** 41** to enhance Tipifarnib efficacy in MDR cancer cells as it reduced the IC_50_ for Tipifarnib from 9.0 *μ*M (applied alone) to 3.8 *μ*M. Activity was compared to Tipifarnib employing human sensitive non-small cell carcinoma cell line NCI-H460 (IC_50_ = 198 *μ*M for compound** 41** alone), its multidrug resistant (MDR) counterpart NCI-H460/R (IC_50_ = 172 *μ*M for compound** 41** alone), and spontaneously immortalized normal human keratinocyte cell line HaCaT. The concentration around 100 *μ*M of compound** 41** significantly inhibited the growth of normal HaCaT cells, considering that HaCaT are normal, but transformed cells with immortalized phenotype; these results point to the potential of compound** 41** to exert the cancer preventive effects. Compound** 41** increased the expression of HIF-1*α*, probably by exerting antioxidant and redox status modulator effect. Compound** 41** increased the expression of HIF-1*α* and acted synergistically with Tipifarnib probably by inhibiting the MAP kinase prosurvival signaling [47]. To identify potent anticancer agents against the cisplatin-resistant human ovarian cancer cell line A2780/Cis, a series of compounds were synthesized by Shin et al. (2014). Among them, the two most active compounds were** 42** (GI_50_ = 3.87 *μ*M) and** 43** (GI_50_ = 3.95 *μ*M) (Figure 14). It was found that compounds** 42** and** 43** trigger cell cycle arrest at the G_2_/M phase and apoptotic cell death in A2780/Cis cancer cells. The compounds act through caspase-7 activation. Aurora A kinase is known to be involved in cell cycle arrest at the G_2_/M phase.* In vitro* aurora A kinase assay of compound** 42** showed the half-maximal inhibitory concentration (IC_50_) of 66.4 *μ*M. Because aurora A kinase may not be a real molecular target of compound** 42**, GI_50_ value of compound** 42** is higher than the IC_50_ value of** 42** [48].

## 4. Conclusion

Chalcone architecture linked with various substitutions, such as methoxy, amino, and hydroxyl, and alteration of both rings has been found to be effective in making it a potential candidate in the anticancer armamentarium. The related studies showed excellent cytotoxic activities of chalcones in the different cell death pathways, not just acting in blocking the process of cell division. Inhibitory concentrations in nanomolar range were impressive to show its ability to arrest cell division. Furthermore, besides preclinical study, clinical study of chalcone may yield the development of research in this field. It is expected that this review may give the medicinal chemists a basis to get in touch with the recent updates and will be helpful for enrichment in this field.
